# Stent Graft Placement by Pseudoaneurysm of the Hepatic Arteries: Efficacy and Patency Rate in Follow-up

**DOI:** 10.1007/s00270-021-02993-0

**Published:** 2021-11-03

**Authors:** F. Pedersoli, V. Van den Bosch, P. Sieben, E. Barzakova, M. Schulze-Hagen, P. Isfort, S. Keil, G. Wiltberger, C. K. Kuhl, P. Bruners

**Affiliations:** 1 Department of Diagnostic and Interventional Radiology, University Hospital RWTH Aachen, Pauwelsstraße 30, 52074 Aachen, Germany; 2 Department of General, Visceral, and Transplantation Surgery, University Hospital of RWTH Aachen, Pauwelsstraße 30, 52074 Aachen, Germany

**Keywords:** Hepatic artery, Stents, Aneurysm, False, Hemorrhage

## Abstract

**Purpose:**

To investigate efficacy and patency status of stent graft implantation in the treatment of hepatic artery pseudoaneurysm.

**Materials and Methods:**

A retrospective analysis of patients who had undergone endovascular treatment of hepatic artery pseudoaneurysms between 2011 and 2020 was performed. Medical records were examined to obtain patients’ surgical histories and to screen for active bleeding. Angiographic data on vascular access, target vessel, material used and technical success, defined as the exclusion of the pseudoaneurysm by means of a stent graft with sufficient control of bleeding, were collected. Vessel patency at follow-up CT was analyzed and classified as short-term (< 6 weeks), mid-term (between 6 weeks and 1 year), and long-term patency (> 1 year). In case of stent occlusion, collateralization and signs of hepatic hypoperfusion were examined.

**Results:**

In total, 30 patients were included and of these, 25 and 5 had undergone stent graft implantation and coiling, respectively. In patients with implanted stent grafts, technical success was achieved in 23/25 patients (92%). Follow-up CT scans were available in 16 patients, showing stent graft patency in 9/16 patients (56%). Short-term, mid-term, and long-term short-term stent patency was found in 81% (13/16), 40% (4/10), and 50% (2/4). In patients with stent graft occlusion, 86% (6/7) exhibited maintenance of arterial liver perfusion via collaterals and 14% (1/7) exhibited liver abscess during follow-up.

**Conclusion:**

Stent graft provides an effective treatment for hepatic artery pseudoaneurysms. Even though patency rates decreased as a function of time, stent occlusion was mainly asymptomatic due to sufficient collateralization.

## Introduction

Pseudoaneurysms of the hepatic arteries represent a rare but potentially fatal complication after major pancreatic or hepatic surgery. Their incidence is increased in complicated postoperative courses with anastomotic fistulae [1]. Because of the high risk of rupture and consequent massive hemorrhage [2], with an associated mortality rate of up to 60% [3], pseudoaneurysms should be treated promptly. Besides surgical vessel repair, endovascular therapies such as stent graft placement or coil embolization [4] are part of the therapeutic algorithm in these patients. Among the interventional and endovascular options, treatment of pseudoaneurysms by stent graft implantation has the advantage of preserving arterial hepatic perfusion, which decreases the onset of major postoperative complications in these patients [5]. Despite preliminary evidence on the efficacy of stent grafting in the treatment of hepatic pseudoaneurysms, clinical studies with substantial patient numbers and meaningful follow-up periods are scarce [6–8].

Therefore, the aim of the present study was to evaluate the efficacy of stent graft placement in the treatment of hepatic artery pseudoaneurysms in a larger cohort of patients with a particular focus on stent graft patency as a function of time.

## Material and Methods

Approval for this retrospective cohort study was waived by the institutional review board (EK 200/21). All consecutive patients who had undergone endovascular treatment for pseudoaneurysms of the hepatic artery from 2011 to 2020 were included. Electronic medical records were examined to collect patient data, history of previous surgeries, primary disease treated surgically, anastomotic leakage and drainage placement, time lapse between surgery and pseudoaneurysm treatment, active bleeding at the diagnosis of pseudoaneurysm and the length of the hospital stay. Active bleeding was defined based on the presence of fresh blood in the surgical drainage, active arterial bleeding shown on contrast-enhanced CT or presentation of patients in hypovolemic shock.

Available CT image datasets acquired before the intervention were reviewed and the diameter of the target vessel was measured in the CT performed immediately before the intervention and, if active bleeding was present at the immediate pre-interventional CT, in the latest, yet earlier CT scan (with the patient clinically stable), to determine the diameter of the artery to exclude the potential influence of vasoconstriction present in hypovolemic shock. CT scans were performed with image acquisition in non-enhanced, arterial, and portal-venous phases. The following angiographic data were collected: vascular access, type of introducer sheath, vessel which was embolized or coiled, involvement of the origin of the gastroduodenal artery, catheters and guidewires used, and embolization material used. For stent grafting, technical success was defined as the exclusion of the pseudoaneurysm through stent graft implantation with control of the eventual bleeding. As a secondary goal, the maintenance of a regular blood flow to the liver arteries on final angiogram was evaluated.

### Imaging Follow-up

Data regarding complications potentially related to stent implantation or stent occlusion, recurrence of bleeding after treatment, readmission for biliary sepsis and mortality were collected. Findings present at follow-up imaging performed by CT were classified as pertaining to the short-term (< six weeks), mid-term (six weeks – one year), and long-term (> one year) follow-up periods. Patency of stent graft and any complications possibly related to hypoperfusion of the liver were assessed. In case of stent thrombosis, patency of the right and left hepatic arteries through collaterals were examined.

### Statistics

Continuous variables are expressed as median and 25th and 75th percentiles (defined as the interquartile range [IQR]) and the 30-day mortality rate was calculated. SPSS 27 (IBM, Armonk, New York, USA) was used for the statistical analysis.

## Results

### Patient Population

Endovascular treatment of hepatic artery pseudoaneurysms was performed in 30 patients (24 males, 6 females) with a median age of 64 years (IQR: 53; 73 years). Previous pancreaticobiliary surgery had been performed in 28/30 cases, with a median time lapse of 18 days (IQR: 9; 39 days) between the surgery and the diagnosis of hepatic pseudoaneurysm. Vessel erosion caused by perihilar cholangiocarcinomas was the cause of pseudoaneurysm in the remaining 2/30 patients. In patients who had undergone pancreaticobiliary surgery, pancreatic fistulae grade B or C according to the International Study Group of Pancreatic Surgery classification [9] were detected in 12 patients. Leakage of the biliodigestive anastomosis, diagnosed by the presence of biliary enzymes following drainage of an extrahepatic fluid collection, was observed in 5 patients. An active bleeding at the time of diagnosis was present in 25/30 patients, whereas hepatic pseudoaneurysms were detected as incidental findings in CT in 5/30 patients. Median length of hospitalization was 21 days (IQR: 15; 50 days). Further, information on the patients’ surgical history is given in Table 1.

**Table 1:** Information on surgical anamnesis of patients

**Previous surgeries**	**28**
Pylorus-preserving pancreaticoduodenectomy	18
Whipple procedure (pancreaticoduodenectomy)	2
Liver transplantation	2
Pylorus-preserving pancreaticoduodenectomy and right hemihepatectomy	1
Right extended hepatectomy	1
Left extended hepatectomy	3
Left pancreatic resection	1
**Underlying disease**	**28**
Pancreatic carcinoma	11
Perihilar cholangiocarcinoma (Klatskin tumor)	6
Necrotizing pancreatitis	4
Intraductal papillary mucinous neoplasm	1
Duodenal carcinoma	1
Pancreatic neuroendocrine tumor	1
Ampullary tumor	1
Hepatitis B	1
Secondary sclerosing cholangitis	1
Iatrogenic duodenal perforation	1
**Leakage**	**16**
Pancreaticojejunostomy	12
Biliodigestive anastomosis	4
**Drainage**	**16**
CT-guided	13
Surgically placed	2
Percutaneous transhepatic biliary	1

### Endovascular Treatment

Treatment was performed directly after detection of the pseudoaneurysm under general anesthesia in 21/30 patients and in local anesthesia in 9/30 patients. A transfemoral access was used in 25 patients and a transbrachial left access in the remaining five patients, with a minimum size of the introducer sheath of 5F (Cook, Bloomington, USA; OSCOR Inc ©, Palm Harbor, Florida, USA; Teleflex Incorporated, Wayne, USA). Then, a 4F or a 5F catheter was advanced in the celiac artery (for transfemoral access: Cobra 2, Cook or Shepherd Hook, Merit Medical, South Jordan, UT, USA; for transbrachial access: Vertebral, Merit Medical). The target vessel was the origin of the gastroduodenal artery between the common and the proper hepatic artery in 15/30 patients, the common hepatic artery in 7/30 patients, the proper hepatic artery in 3/30 patients, the right hepatic artery in 4/30 patients and both the left hepatic artery and an aberrant right hepatic artery originating from the superior mesenteric artery in 1/30 patient. Pseudoaneurysms arising proximal to the hepatic bifurcation (25/30 patients) were treated by stent graft implantation. Coil embolization was performed with pseudoaneurysms of the left or right hepatic artery (5/30 patients).

### Stent Graft Implantation

**Fig. 1:**
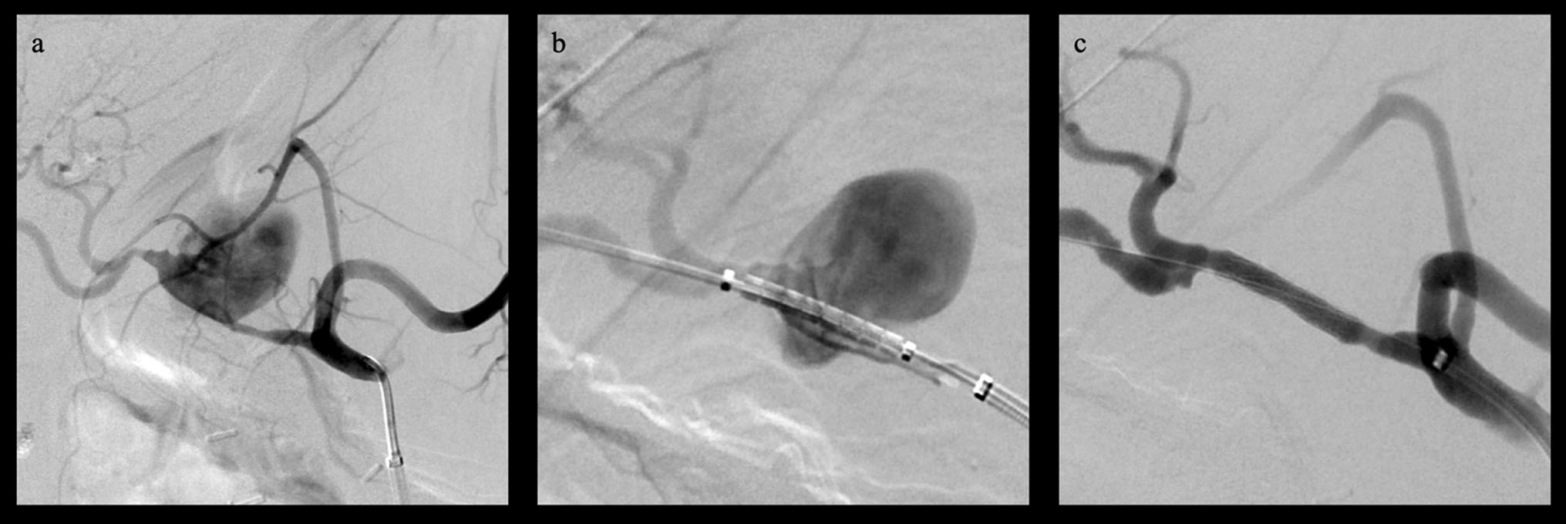
Digital subtraction angiograms demonstrate a large pseudoaneurysm of the hepatic artery in this 63-year-old patient 46 days after pylorus-preserving pancreaticoduodenectomy performed to treat necrotizing pancreatitis (**a**). A 5 × 25 mm stent graft (VIABAHN®, W. L. Gore & Associates, Inc. Flagstaff, USA) was placed across the pseudoaneurysm neck to exclude the pseudoaneurysm, before (**b**) and after deployment (**c**)

A stiff 0.014″ or 0.018″ guidewire (Spartacore or Steelcore, Abbott, Abbott Park, USA) was placed distal to the pseudoaneurysm with a 2.4F or a 2.7F microcatheter (Progreat, Terumo Medical Corporation, Tokyo, Japan). A self-expandable or balloon-expandable stent graft was then positioned across the neck of the pseudoaneurysm (Fig. 1). The selection of the stent type was based on the individual experience of the interventionalist; self-expandable stent grafts were generally preferred in the case of larger, more straight vessels and more proximally located pseudoaneurysms, whereas balloon-expandable stent grafts were used in peripheric or tortuous vessels. Technical details of the stent grafts employed are reported in Table 2. Overall, the dimension of the stents was comparable to the dimension of the target vessel in CT scans performed in clinically stable conditions, with a median difference of 0 mm (IQR: − 0.5 mm; 0 mm) and oversized compared to CT performed directly before the intervention with a median difference of + 1 mm (IQR: 1 mm; 2 mm). When compared to the angiogram the difference was + 2 mm (IQR: 1 mm; 2 mm).

**Table 2:** Features of stent grafts used and numbers of patients. Stent used: VIABAHN® (W. L. Gore & Associates, Inc. Flagstaff, USA), Advanta V12 (Atrium/Maquet Cardiovascular, Hudson, USA), and BeGraft, (Bentley InnoMed GmbH, Hechingen, Germany)

Stent model	
6 × 50 mm VIABAHN®	5
6 × 25 mm VIABAHN®	6
7 × 25 mm VIABAHN®	2
5 × 25 mm VIABAHN®	2
8 × 38 mm Advanta V12	1
8 × 25 mm VIABAHN®	1
7 × 50 mm VIABAHN®	1
5 × 50 mm VIABAHN®	1
5 × 22 BeGraft	1
4 × 16 BeGraft	1
4 × 24 BeGraft	1
8 × 50 VIABAHN® 2 overlapping	1
6 × 25 mm VIABAHN® + 5 × 25 mm VIABAHN®	1
5 × 22 Advanta V12 + 6 × 25 mm VIABAHN®	1

A bolus of 5000 IU heparin was administered during the intervention; partial thromboplastin time (PTT)-based heparinization (with a target range of 40–60 s) was continued for 24 h after the intervention. Oral administration of 100 mg/day aspirin was initiated after the intervention and was prescribed as a lifelong medication, whereas clopidogrel 75 mg/day was started after clinical stabilization and continued for six weeks.

**Fig. 2:**
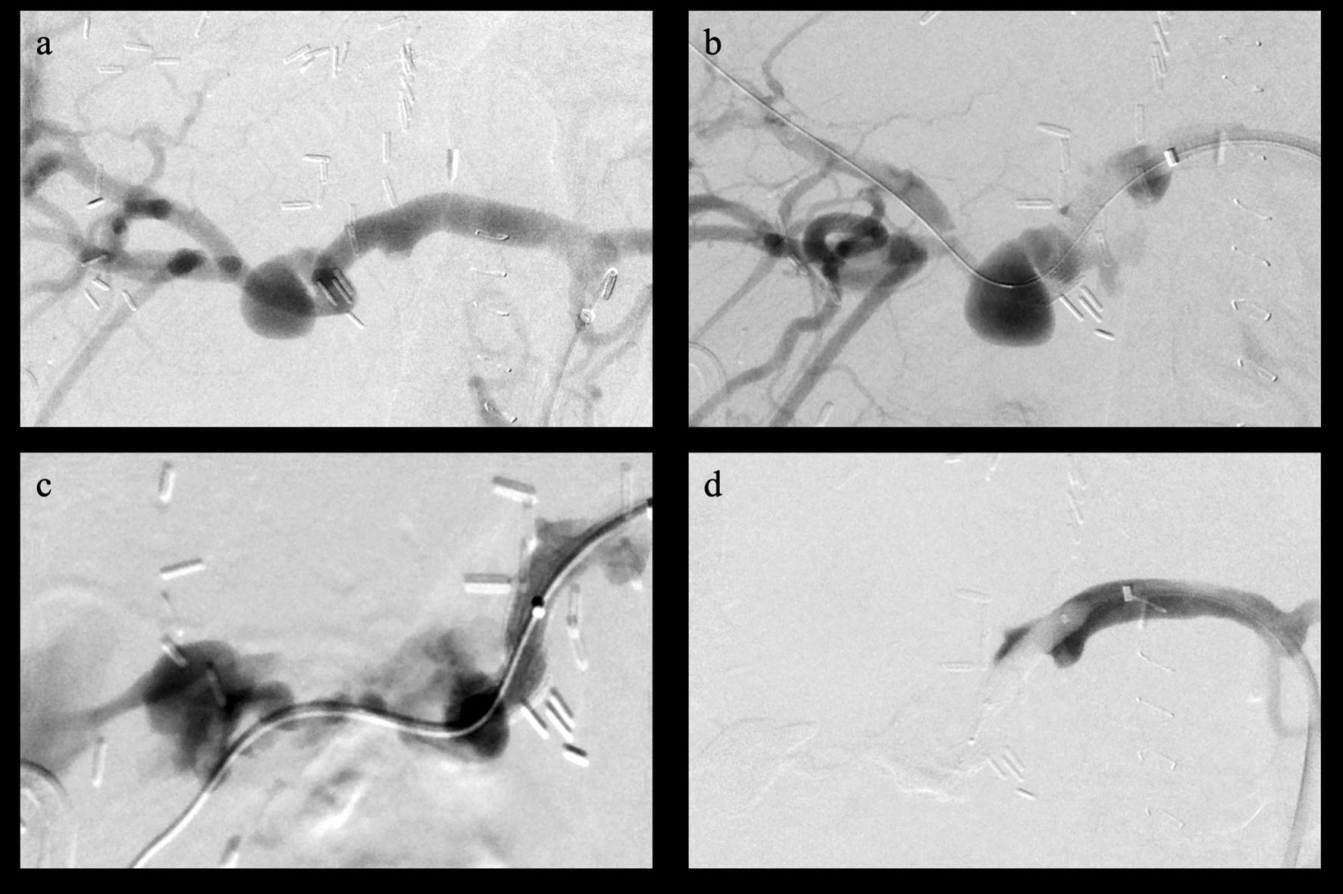
Digital subtraction angiography demonstrates a moderately sized pseudoaneurysm of the proper hepatic artery (**a**). Incomplete coverage of the pseuroaneurysm neck with a 6 × 25 mm stent graft (VIABAHN®, W. L. Gore & Associates, Inc. Flagstaff, USA) with residual perfusion of the pseudoaneurysm (**b**). Vessel rupture with contrast media extravasation distant to the distal end of the stent (**c**) after the attempted deployment of a 5 × 25 mm stent graft (VIABAHN®). The bleeding was immediately blocked with a 5 × 20 mm balloon catheter (Armada, Abbott, Abbott Park, USA), inflated with saline solution (**d**), and the patient was immediately transferred to open surgery where the complete rupture of the vessel was confirmed

Technical success was achieved in 23/25 cases (92%). Stent deployment was not possible during the first intervention in 1/30 patients because of the tortuous anatomy of the proper hepatic artery; since the patient was clinically stable without active bleeding, a second successful attempt was performed the day after. A complete rupture of the common hepatic artery occurred in 1/30 cases. A balloon catheter was then inflated within the artery to stop the bleeding and emergency surgical treatment was successful (Fig. 2). Hemorrhage was stopped in every patient (20/20) who presented with active bleeding at the time of diagnosis.

Blood flow in the hepatic artery was maintained in 22/25 patients; complete stent thrombosis refractory to aspiration and local thrombolysis was observed in 1/25 patient. A dissection in the left or the right hepatic artery was detected in three patients; this was not considered to be flow limiting as it was not associated with a delay in the enhancement of liver parenchyma in DSA.

In two patients, a feeder vessel from the gastroduodenal artery which supplied the pseudoaneurysm was successfully embolized using coils (Hilal or Nester, Cook) after stent deployment via the gastroduodenal arcade. An actively bleeding endoleak type 1a was successfully treated by means of stent graft extension in one patient six days after treatment.

### Coil Embolization

Embolization with coils (2–4 mm Hilal or Nester, Cook) of the right or left hepatic artery was performed in 5/30 patients. Complete vessel embolization was observed in 5/5 cases (100%).

### Clinical Follow-up (Stent Graft)

30-day mortality was 28% (7/25 patients). Cause of death was multiorgan insufficiency, including liver insufficiency, in five patients, and septic shock and cardiogenic shock in one patient each. The patient who died because of septic shock had a biliary sepsis after undergoing left extended hepatectomy and biliodigestive anastomosis. The patient who died because of cardiogenic shock did so during surgery performed for abdominal rebleeding seven days after stent implantation. This patient was directly operated on without performing another CT scan and the source of bleeding could not be identified. Among these patients, the stent was patent in 3/7 patients, stent occlusion was present in 1/7 patients, while 3/7 patients had no follow-up imaging data available. No patients died because of complications related to the stent graft implantation. Readmission because of biliary sepsis was observed in 5/25 patients.

### Imaging Follow-up (Stent Graft)

**Fig. 3:**
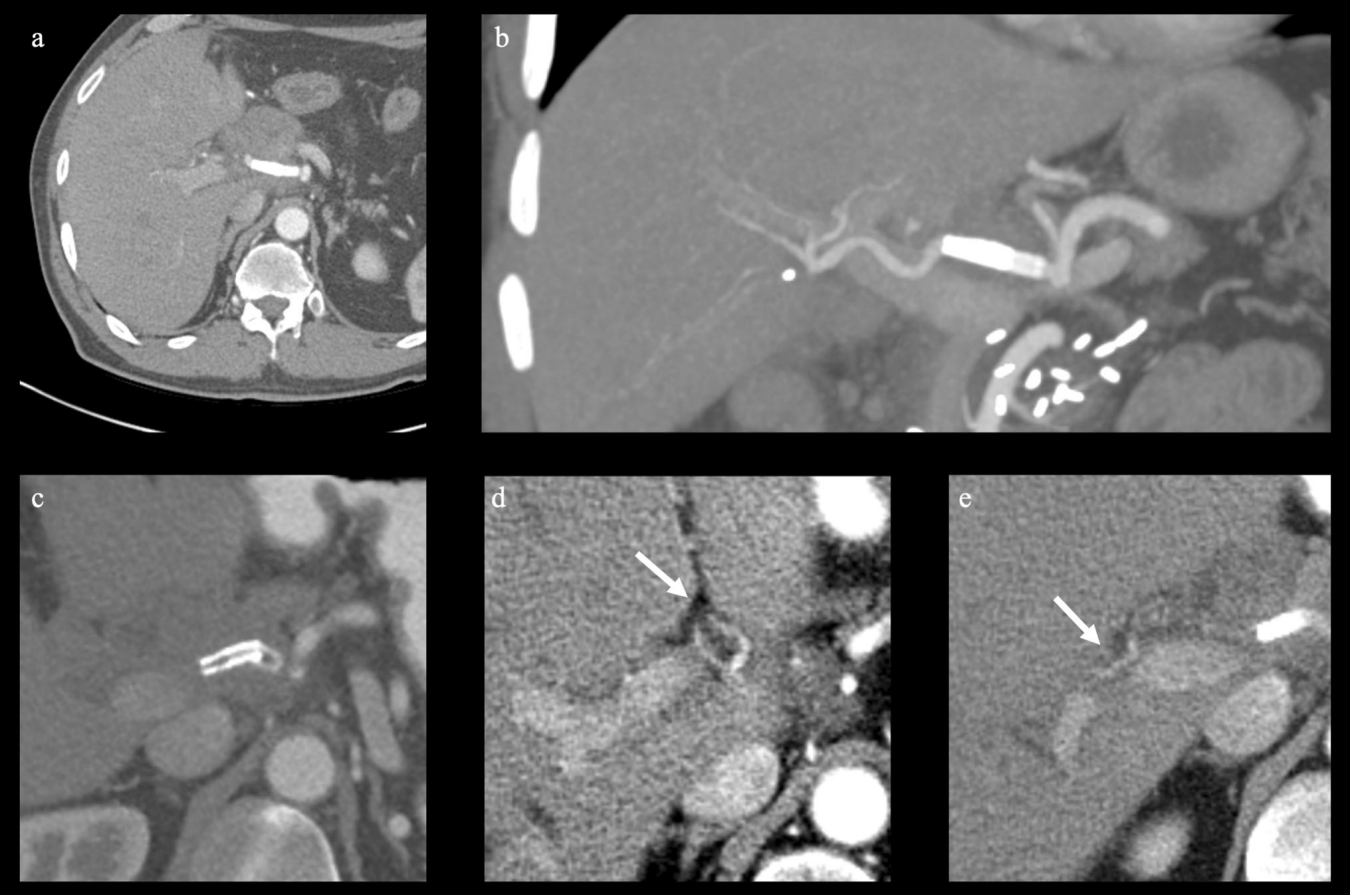
Early follow-up with CT scans in axial reconstruction (**a**) and coronal maximum intensity projection (**b**) 20 days after implantation of two stent grafts (proximal 6 × 25 mm, distal 5 × 25 mm VIABAHN®, W. L. Gore & Associates, Inc. Flagstaff, USA) with patency of the stents. Long-term follow-up 468 days after stent implantation showing stent thrombosis (**c**) with patency of the left (**d**) and right (**e**) hepatic arteries (arrows). Images **c**, **d**, and **e** are axial reconstructions

Imaging follow-up was available in 16/25 patients who had undergone stent graft implantation, with a median of 63 days (IQR: 10; 396), showing stent graft patency in 9/16 patients and occlusion in 7/16 patients. Short-term follow-up imaging data (< six weeks after the intervention) were available in 16 patients and showed a patency rate of 13/16 patients at a median of 14 days after the intervention (IQR: 5; 25). Mid-term follow-up imaging data (between six weeks and one year), available in 10 patients, showed a patency rate of 4/10 at a median of 187 days after the intervention (IQR: 91; 257). Long-term follow-up imaging data (> one year), available in four patients, showed a patency rate of 2/4 at a median of 1700 days (IQR: 1183; 2009). In patients with stent occlusion, the left and right hepatic arteries were both perfused via collaterals in 6/7 patients (Fig. 3). 1/7 patients presented with liver abscess at follow-up, which may also be related to stent occlusion; this patient underwent biliodigestive anastomosis after left extended hepatectomy. Beyond that, no signs of reduced or absent liver perfusion were present at follow-up.

## Discussion

Stent graft implantation was an effective treatment for pseudoaneurysms of the hepatic artery, with a technical success rate of 92% in pseudoaneurysm exclusion, with maintenance of the hepatic arterial flow in 88% cases. High stent patency rates (81%) were observed at short-term follow-up with a decrease to 40% at mid-to-long-term follow-up.

These encouraging results on the clinical efficacy of stent grafting in this context are in line with current literature data, with described technical success higher than 85% [10–13]. In contrast to the homogeneous results on clinical efficacy, reported stent patency at follow-up varies considerably, from 42 to 100%. Hassold et al. reported stent patency rates of 84% 30 days after implantation with a decrease to 42% after one year^8^, comparable to our findings.

Other studies reported only the patency rates of the last control imaging. The results of the most relevant studies are summarized in Table 3.

**Table 3:** Results of the most important literature studies (> 5 patients). Ultrasound (US); computer tomography (CT)

	Number of patients [n]	Stent model	Technical success rate	Rebleeding [n]	Patency rates	Follow-up duration and type of imaging used
Bellemann et al	24	Balloon expandable	88%	2	89%	4 months (CT and US)
Cui et al	17	Balloon and self-expandable	100%	4	100%	23 months (US)
Wang et al	9	Balloon expandable	100%	2	100%	10 months (US)
Lü et al	8		100%	0	75%	14 months
Venturini et al	18	Self-expandable	100%	2	x	x
Hassold et al	16	Balloon expandable	88%	1	84%	1 month (CT and angiography)
					42%	12 months (CT and angiography)

Similarly, the anticoagulant and antiplatelet therapy regimes used after stent graft implantation are not homogeneous in the literature, ranging from total absence (to minimize the risk of rebleeding [7]) to Aspirin at a lifelong dosage of 150 mg/day combined with Clopidrogrel at a dosage of 75 mg/day for 12 weeks [10]. According to the guidelines of the European Societies of Cardiology and for Vascular Surgery, there is no recommendation regarding single or dual antiplatelet therapy after endovascular treatments in the visceral arteries; most centers empirically prescribe a combination of daily Clopidogrel (75 mg) and Aspirin (low dose) from one month to one year [14]. Although in our study a direct association between stent occlusion and stop of dual antiplatelet therapy cannot be proven, the concurrence of stent occlusion and the discontinuation of Clopidogrel suggest a potential benefit of longer dual antiplatelet therapy in patients at risk of stent occlusion, as suggested after coronary stent placement [15].

Risk of rebleeding can represent a contraindication for dual antiplatelet therapy after major hepatobiliary surgery in patients with active bleeding. In our cohort, one patient presented with significant abdominal rebleeding which could be possibly related to the anticoagulant and antiplatelet therapy. The causes of death in the other patients were not related to the antiplatelet therapy and bleeding of pseudoaneurysms was part of the process which led to multiorgan failure and contributed to the relevant 30-day mortality. Consequently, dual antiplatelet therapy must be evaluated in consideration of the clinical conditions of patients.

Stent occlusion was mainly asymptomatic and characterized by sufficient collateralization in most patient (86%). High degrees of collateralization were reported previously by Hur et al. with maintenance of hepatic arterial flow in 10/13 patients after coiling of the hepatic artery [16]. These results may justify coiling in case of tortuous or small vessels. In our cohort, stent deployment was possible in 23/25 patients as the pseudoaneurysm was located proximal to the hepatic bifurcation. In consideration of possible ischemia after coiling [17], a stent grafting of the proximal hepatic artery should always be attempted first before resorting to coiling.

Limitations of this study are represented by its retrospective and monocentric design, which, together with the low incidence of this complication, limits the collection of a larger and more varied patient population. Another limitation is the absence of standardized follow-up CT studies which were not available 9/25 patients due to variable referral practice; however, current evidence does not suggest performing CT imaging in asymptomatic patients; moreover, the inherently poor prognosis of these patients hampers the collection of substantially larger patient sizes at mid- and long-term.

In conclusion, stent graft placement was confirmed to be an effective treatment for hepatic artery pseudoaneurysm, especially in acute bleeding. Patency rates were high in the short term yet decreased substantially as a function of time. If present, stent occlusion was mainly asymptomatic due to the development of collateral vessels which provided sufficient hepatic blood supply.
